# Long-term follow-up of patients after acute kidney injury in the neonatal period: abnormal ambulatory blood pressure findings

**DOI:** 10.1186/s12882-022-02735-5

**Published:** 2022-03-23

**Authors:** Gulsen Akkoc, Ali Duzova, Ayse Korkmaz, Berna Oguz, Sule Yigit, Murat Yurdakok

**Affiliations:** 1 Department of Pediatric Infectious Disease, University of Health Sciences, Haseki Training and Research Hospital Istanbul, Istanbul, Turkey; 2 Division of Pediatric Nephrology, Faculty of Medicine, Hacettepe University, Ankara, Turkey; 3 Section of Neonatology, Department of Pediatrics, School of Medicine, Acıbadem University, Istanbul, Turkey; 4 Department of Radiology, Faculty of Medicine, Hacettepe University, Ankara, Turkey; 5 Division of Neonatology, Faculty of Medicine, Hacettepe University, Ankara, Turkey

**Keywords:** Long-term follow-up, Acute kidney injury, Neonate, Hyperfiltration, Microalbuminuria, Ambulatory blood pressure monitoring

## Abstract

**Background:**

Data on the long-term effects of neonatal acute kidney injury (AKI) are limited.

**Methods:**

We invited 302 children who had neonatal AKI and survived to hospital discharge; out of 95 patients who agreed to participate in the study, 23 cases were excluded due to primary kidney, cardiac, or metabolic diseases. KDIGO definition was used to define AKI. When a newborn had no previous serum creatinine, AKI was defined as serum creatinine above the mean plus two standard deviations (SD) (or above 97.5^th^ percentile) according to gestational age, weight, and postnatal age. Clinical and laboratory features in the neonatal AKI period were recorded for 72 cases; at long-term evaluation (2–12 years), kidney function tests with glomerular filtration rate (eGFR) by the Schwartz formula, microalbuminuria, office and 24-h ambulatory blood pressure monitoring (ABPM), and kidney ultrasonography were performed.

**Results:**

Forty-two patients (58%) had stage I AKI during the neonatal period. Mean age at long-term evaluation was 6.8 ± 2.9 years (range: 2.3–12.0); mean eGFR was 152.3 ± 26.5 ml/min/1.73 m^2^. Office hypertension (systolic and/or diastolic BP ≥ 95^th^ percentile), microalbuminuria (> 30 mg/g creatinine), and hyperfiltration (> 187 ml/min/1.73 m^2^) were present in 13.0%, 12.7%, and 9.7% of patients, respectively. ABPM was performed on 27 patients, 18.5% had hypertension, and 40.7% were non-dippers; 48.1% had abnormal findings. Female sex was associated with microalbuminuria; low birth weight (< 1,500 g) and low gestational age (< 32 weeks) were associated with hypertension by ABPM. Twenty-three patients (33.8%) had at least one sign of microalbuminuria, office hypertension, or hyperfiltration. Among 27 patients who had ABPM, 16 (59.3%) had at least one sign of microalbuminuria, abnormal ABPM (hypertension and/or non-dipping), or hyperfiltration.

**Conclusion:**

Even children who experienced stage 1 and 2 neonatal AKI are at risk for subclinical kidney dysfunction. Non-dipping is seen in four out of 10 children. Long-term follow-up of these patients is necessary.

## Background

The incidence of acute kidney injury (AKI) in newborns is high compared to other age groups of childhood. Its incidence is 20–40% in neonatal intensive care units (NICUs) [1–3]; low birth weight, patent ductus arteriosus (PDA), and non-steroidal anti-inflammatory drug (NSAID) use are associated with the development of AKI [1, 4–6]. In neonates, prognosis and recovery from AKI depend on the underlying cause [6, 7]. Meta-analyses in adults have found a significant correlation between AKI and chronic kidney disease (CKD) as well as end-stage kidney disease (ESKD) [8, 9]. In a pediatric series reported by Askenazi et al., 174 patients were followed up 3 to 5 years after AKI in childhood; 32 of them died, and among 29 patients who accepted re-evaluation 31%, 14%, 28%, and 14% of patients had stage 1 CKD, stage 2 CKD, microalbuminuria, and hypertension, respectively [10]. Sinha et al. showed that six (38%) out of 16 patients, who had AKI between 1 month and 10 years of age, had at least one of the following 10 years later: hypertension or proteinuria/hematuria or abnormal creatinine level [11]. Mammen et al. revealed that 10% of 126 patients who had AKI in childhood (including 30 cases younger than 28 days) developed CKD 1–3 years after AKI [12]. In a retrospective study of critically ill children who had AKI at 6.8 ± 6.4 years of age (range: 0 days to 18 years), 32 of 474 patients had progression to CKD or ESKD in 2–4 years [13].

There is limited knowledge about the long-term effects of neonatal AKI, such as hypertension, decreased kidney function, and tubular malfunction. Kidney dysplasia, obstructive uropathy, cortical necrosis, and cystic kidney diseases are risk factors for progression to CKD in the future [14]. In addition, animal studies revealed that AKI secondary to hypoxic, ischemic and nephrotoxic causes results in a low number of peritubular capillaries, a decrease in kidney function in subsequent years, and progression to CKD [15–18]. After a follow-up period of 8 years, 20 extremely low birth weight preterm infants who had suffered from AKI had all suffered from proteinuria and impaired kidney function; renovascular hypertension, CKD and ESKD were seen in two, four, and five patients, respectively [19].

We aimed to assess the prevalence of long-term (2–12 years) kidney dysfunction (microalbuminuria, hypertension, hyperfiltration), based on clinical indicators including ambulatory blood pressure monitoring (ABPM), and to determine associated factors in a case series of children who had suffered from AKI in the neonatal period (excluding primary kidney, cardiac, or metabolic diseases).

## Methods

### Setting and participants

Medical records of patients hospitalized in newborn wards of Hacettepe University Ihsan Dogramaci Children’s Hospital from January 2000 to December 2009 were retrospectively reviewed to identify the patients who suffered from AKI. AKI was defined as an increase in serum creatinine level of 0.3 mg/dl or more within 48 h or 50% or more from the previous lowest value within 7 days, based on the Kidney Disease: Improving Global Outcomes (KDIGO) workgroup’s definition of AKI modified for neonates, as used in previous neonatal studies [20–22]. We excluded urine output since we were not able to check hourly urine output in all cases. When a newborn had no previous serum creatinine, AKI was defined as serum creatinine above the mean plus two standard deviations (SD) (or above 97.5^th^ percentile) according to birth characteristics (gestational age and weight) and postnatal age [23–25].

**Fig. 1:**
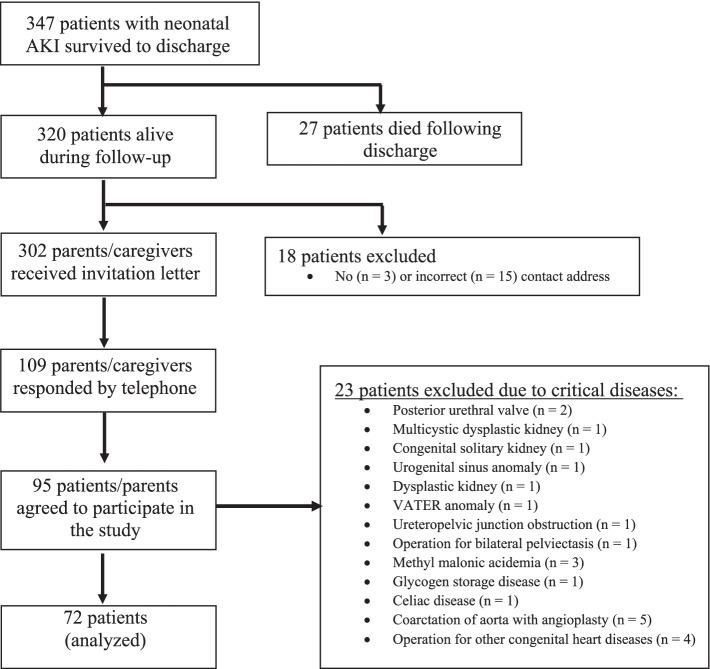
Diagram showing the selection of patients in the study. In total, 72 subjects were analyzed for signs of long-term kidney dysfunction

Of the patients who had developed neonatal AKI according to the defined criteria, 347 patients survived and were discharged. Of those, 27 patients (7.8%) had died following discharge. Of 302 parents or caregivers who were sent invitation letters, 109 responded by telephone, and 95 agreed to participate in the study. Twenty-three patients with primary kidney, cardiac or metabolic diseases that may affect long-term evaluation were excluded. In total, 72 patients were analyzed, including 46 males (63.9%) and 26 females (36.1%) (Fig. 1).

### Clinical data from neonatal period

Clinical data were retrospectively recorded, including date of birth, gender, gestational age, birth weight, prenatal features, medical conditions before AKI (e.g., systemic diseases, respiratory distress syndrome [RDS], PDA, necrotizing enterocolitis [NEC], disseminated intravascular coagulation [DIC], inherited metabolic diseases, congenital heart disease, surgical procedures, drugs, mechanical ventilation, exposure to radio-contrast), AKI features (age at AKI, signs and symptoms at the time of diagnosis, laboratory results, diuretic or inotrope use, indication and duration of kidney replacement therapy [KRT]). AKI severity was classified according to KDIGO methods.

### Re-evaluation of patients

Patients were asked about their current medical condition, chronic diseases, and medications. Physical examination, weight, height, body mass index (BMI) measurement; laboratory studies including complete blood count, kidney function test, venous blood gas, urinalysis, urinary microalbumin, creatinine, phosphate; kidney ultrasonography (US), kidney Doppler US, auscultatory office blood pressure (BP) measurement, and 24-h ABPM were performed.

### Definitions

Serum creatinine was measured with Jaffe method (alkaline picrate). *Estimated glomerular filtration rate* (eGFR) was calculated based on the Schwartz formula [26].

*Hyperfiltration:* Mean GFR between 2 to 12 years of age is 133 ± 27 ml/min/1.73 m^2^ [28, 29]. GFR values above 187 ml/min/1.73 m^2^ (> mean + 2 SD) [26, 27].

*Microalbuminuria* was defined as a urinary microalbumin (mg)/creatinine (g) ratio (ACR) above 30 in a spot urine sample.

*Tubular phosphorus reabsorption (TPR)* was calculated; *TmP/GFR* was defined as maximum serum phosphorus level adjusted to GFR and measured as TmP/GFR = TPR x serum phosphorus level. The normal range is 4–⁠6 mg/dl.

*Tubular function disorder* was defined as TPR level below 85% and/or venous blood HCO_3_ level below 20 mmol/L.

### Office and ambulatory blood pressure measurement

Standard procedures were applied for office BP measurement using the auscultatory method; BP was measured three times with an interval of 3 min between measurements and the average of the last two was used. The patient’s BP percentile was determined according to age, gender and height percentile according to 2016 European Society of Hypertension guidelines [28]. The presence of systolic and/or diastolic BP ≥ 95^th^ percentile was defined as hypertension.

24-h ABPM was performed in patients with height ≥ 120 cm and ≥ 6 years of age; patients with poor cooperation or who were not able to carry the equipment for 24 h were excluded. Patients were not hospitalized during ABPMs and they performed normal daily activities. Spacelabs ABPM devices (Model no: 90207–30) were used. Measurements were performed every 15–20 min during waking hours (day) and every 30 min during sleep periods (night). A valid ABPM profile was defined as follows: 24-h recording with at least 70% of expected measurements, at least 40 to 50 readings for a 24-h period; and at least two valid daytime and one valid nighttime measurement per hour [29, 30]. The mean systolic BP (SBP), diastolic BP (DBP) and arterial pressure (MAP) levels and load (percentage of readings above the ambulatory 95^th^ percentile by sex and age) were calculated for the 24-h period, daytime (from 08.00 am to 08.00 pm), and nighttime (from midnight to 06.00 am) periods. SD scores (SDS) for SBP, DBP and MAP were determined according to the standard values adjusted by age and gender [31]. The presence of SBP or DBP or MAP ≥ 95 percentile; or the presence of BP load ≥ 50% was defined as ABPM hypertension. Dipping was defined based on the percent decline in mean systolic or diastolic levels from daytime to nighttime (100 x [mean daytime – mean nighttime] / [mean daytime]); a dipping level < 10% was defined as non-dipping. White-coat hypertension was defined as office BP ≥ 95^th^ percentile, with normal ABPM profile. Masked hypertension was defined as office BP < 95^th^ percentile and ABPM hypertension [29].

### Kidney and Doppler US

Patients underwent kidney US and kidney Doppler US in the supine position. All evaluations were performed by a single pediatric radiologist (BO). Kidney size, parenchymal thickness, parenchymal echogenicity, and the kidney Doppler indices (resistive index, acceleration and acceleration time) were evaluated by US. Kidney vertical lengths were evaluated according to age, sex, weight, and height, and percentiles were determined [32].

### Long-term kidney dysfunction sets

Long-term kidney dysfunction was defined as the presence of at least one sign of microalbuminuria (ACR > 30), hypertension (office or ABPM), or hyperfiltration (eGFR > 187 ml/min/1.73 m^2^). Three kidney dysfunction sets (KDS) were defined for the evaluation. KDS-1 was defined as the presence of microalbuminuria and/or hypertension by office BP and/or hyperfiltration (eGFR > 187 ml/min/1.73 m^2^); KDS-2 was defined as the presence of microalbuminuria and/or hypertension with ABPM and/or hyperfiltration. KDS-3 was defined as the presence of microalbuminuria and/or abnormal ABPM (hypertension and/or non-dipping) and/or hyperfiltration. Univariate analysis was used to evaluate the following as independents factors for the presence of proteinuria, hypertension, hyperfiltration, KDS-1, KDS-2, and KDS-3: age, gender, birth weight, gestational age, weight according to gestational age, systemic diseases in the neonatal period, mechanical ventilation, surgical procedure before AKI, age at AKI diagnosis, total fluid intake before AKI, aminoglycoside use, indomethacin use, radiocontrast use, vasopressor drug use, oliguria, AKI stage, and KRT. Since the significance of hyperfiltration for long-term kidney dysfunction is still unclear; we additionally, repeated our analyses excluding hyperfiltration from the composite outcome.

Non-interventional Clinical Researches Ethics Board of Hacettepe University approved the study. Informed consent was obtained from the parents of the all participants included in the study.

### Statistical analysis

Data analysis was performed using SPSS for Windows 22 package program. In descriptive statistics, the variables with normal distribution were calculated as mean ± SD; the variables without normal distribution were calculated as median (interquartile range, IQR), and the categorical variables were calculated as the number of cases and percentage. The categorical variables were evaluated by Pearson’s chi-square or Fisher’s exact test. Student’s t-test and Mann–Whitney U test were used to determine the significance of the difference between the groups. A p-value of ≤ 0.05 was considered to be statistically significant.

## Results

Baseline clinical features of the patients are summarized in Table 1. The mean gestational age was 34.5 ± 4.7 weeks (range: 24–42 weeks), and the median birth weight of the patients was 2,015 g (IQR 2,045 g). Forty-two (58.3%), 17 (23.6%) and 13 patients (18.1%) had suffered from stage I, II, and III AKI, respectively.

**Table 1:** Baseline clinical characteristics of patients during neonatal period (*N* = 72)

Features	Results
Male/female, n/*n* (%/%)	46/26 (63.9/36.1)
Gestational age, *n* (%)	
24- < 28 weeks	6 (8.3)
28- < 32 weeks	17 (23.6)
32- < 36 weeks	12 (16.7)
36–42 weeks	37 (51.4)
Birth weight, *n* (%)	
500–999 g	9 (12.5)
1,000–1,499 g	12 (16.7)
1,500–2,499 g	15 (20.8)
2,500–3,999 g	34 (47.2)
4,000–5,000 g	2 (2.8)
Small for gestational age	8 (11.1)
Large for gestational age	9 (12.5)
Systemic diseases^a^, *n* (%)	
Sepsis	30 (41.6)
Patent ductus arteriosus	25 (34.7)
Respiratory distress syndrome	20 (27.7)
Cardiac insufficiency	16 (22.2)
Disseminated intravascular coagulopathy	12 (16.7)
Necrotizing enterocolitis	11 (15.2)
Median age at AKI diagnosis (days)	5 (IQR 6)
0–3 days, *n* (%)	14 (19.4)
4–7 days, *n* (%)	36 (50.0)
8–14 days, *n* (%)	11 (15.3)
> 14 days, *n* (%)	11 (15.3)
Before AKI, *n* (%)	
Mechanical ventilation	30 (41.6)
Surgery	8 (11.1)
Aminoglycoside	42 (58.3)
Indomethacin	17 (23.6)
Radio-contrast	2 (2.8)
Vasopressor use, *n* (%)	24 (33.3)
Diuretic use, *n* (%)	22 (30.6)
Median duration of oligo-anuria among 22 patients (days)	2 (IQR 4, range: 1–8)
Kidney replacement therapy (peritoneal dialysis), *n* (%)	7 (9.7)

*AKI* Acute kidney injury

^a^some patients had multiple systemic diseases

The mean age of the participants at the time of assessment was 6.8 ± 2.9 years (range: 2.3–12.0 years). The mean ages of boys (6.5 ± 2.8 years) and girls (7.4 ± 3.0 years) were comparable (*p* = 0.247). Clinical and laboratory characteristics of patients at long-term evaluation are summarized in Table 2. All patients had normal serum creatinine, BUN, and serum electrolyte levels. Mean eGFR was 152.3 ± 26.6 ml/min/1.73 m^2^ (range: 99–229 ml/min/1.73 m^2^). Mean eGFR levels were 154.0 ± 29.3 ml/min/1.73 m^2^ in males and 151.2 ± 21.3 ml/min/1.73 m^2^ in girls (*p* = 0.785), and they were 161.3 ± 30.8 ml/min/1.73 m^2^ between the ages of 2 to ⁠6 and 145.9 ± 21.2 ml/min/1.73 m^2^ between the ages of > 6 to 12 years (*p* = 0.014).

**Table 2:** Clinical and laboratory characteristics of patients at long-term evaluation (*N* = 72)

Features	Results
Age at evaluation / follow-up duration (years)	6.82 ± 2.93 (range: 2.28–12.02)
2—< 6 years, *n* (%)	30 (41.6)
6—13 years, *n* (%)	42 (58.4)
Height-SDS	-0.14 ± 1.17
Short stature, *n* (%)	2 (2.8)
Weight-SDS	-0.02 ± 1.31
Malnutrition, *n* (%)	4 (5.6)
Overweight^a^, *n* (%)	9 (12.5)
Obesity^a^, *n* (%)	10 (13.8)
Laboratory findings	
Blood	
Hemoglobin (g/dl)	12.97 ± 0.81
Anemia, *n* (%)	4 (5.6)
BUN (mg/dl)	12.64 ± 2.74
Creatinine (mg/dl)	0.44 ± 0.11
Uric acid (mg/dl)	3.78 ± 0.91
Sodium (mEq/L)	141.6 ± 1.9
Potassium (mEq/L)	4.52 ± 0.36
Chloride (mEq/L)	105.8 ± 2.0
Calcium (mg/dl)	9.68 ± 0.44
Phosphorus (mg/dl)	4.86 ± 0.68
Magnesium (mg/dl)	2.07 ± 0.17
Serum bicarbonate (mmol/L)	22.6 ± 1.2
eGFR (ml/min/1.73m^2^)	152.3 ± 26.6
> 187 ml/min/1.73m^2^, *n* (%)	7 (9.7)
> 150 ml/min/1.73m^2^, *n* (%)	37 (51.3)
Median ACR (mg/g)	17.5 (IQR 17.3, range: 2.75 – 199.85)
0–30, *n*/*N* (%)	62/71 (87.3)
> 30, *n*/*N* (%)	9/71 (12.7)
Urinary	
TPR (%)	90.3 ± 3.6
TPR < 85%, *n*/*N* (%)	5/70 (7.1)
TmP/GFR (mg/dl)	4.39 ± 0.66
TmP/GFR > 6 mg/dl, *n* (%)	1/70 (1.4)

ACR Urinary albumin (mg) to creatinine (g) ratio, eGFR Estimated glomerular filtration rate, SDS Standard deviation score, TmP (GFR) Maximum serum phosphorus level adjusted to GFR, TPR Tubular phosphorus reabsorption

^a^overweight: BMI is between 85^th^-95^th^ percentile, obesity: BMI ≥ 95^th^ percentile

### Office and ambulatory blood pressure measurements

Office auscultatory BP measurements were available for 69 patients; BP was not measured in three patients due to poor cooperation. Nine patients (13.0%) had either systolic and diastolic (*n *= 8) or only systolic (*n* = 1) BP ≥ 95^th^ percentile.

24-h ABPM measurements were available for 27 patients (6.6–12.0 years). ABPM was not performed in patients who were shorter than 120 cm (*n* = 23), who were not able to stay overnight (*n *= 17), who gave insufficient cooperation (*n *= 4), and who did not accept (*n* = 1).

Detailed results of the 24-h ABPM are presented in Table 3. Five patients (18.5%) had hypertension by ABPM, defined as an elevated BP value (*n* = 4; SBP or DBP or MAP ≥ 95^th^ percentile) or elevated BP load (*n *= 3; SBP or DBP or MAP load ≥ 50%) during 24-h or daytime or nighttime. Eleven patients (40.7%) had systolic (*n* = 10; 37.0%) or diastolic (*n* = 2; 7.4%) non-dipper; only 14 patients (51.9%) had a normal ABPM pattern (normal SDS values, BP load < 25%, and dipper). When combined with office BP measurement, 19 patients (70.4%) had normal BP, three patients (11.1%) had white coat hypertension, four patients (14.8%) had masked hypertension, and one patient (3.7%) had elevated BP with both office BP and ABPM.

**Table 3:** Detailed analysis of 24-h ambulatory blood pressure measurements of 27 patients

**Parameters**	**Periods**		
	**24-h**	**Daytime**	**Night-time**
SBP-SDS	-0.29 ± 1.15	-0.28 ± 1.10	0.12 ± 0.94
≥ 95^th^ p, *n* (%)	2 (7.4)	3 (11.1)	2 (7.4)
90-95^th^ p, *n* (%)	1 (3.7)	-	3 (11.1)
DBP-SDS	-0.37 ± 1.04	-0.45 ± 0.77	0.05 ± 0.94
≥ 95^th^ p, *n* (%)	-	-	2 (7.4)
90-95^th^ p, *n* (%)	2 (7.4)	-	1 (3.7)
MAP-SDS	0.03 ± 1.07	-0.10 ± 0.93	0.46 ± 0.84
≥ 95^th^ p, *n* (%)	3 (11.1)	-	4 (14.8)
90-95^th^ p, *n* (%)	-	3 (11.1)	-
Hypertension with different criteria			
-MAP ≥ 95^th^ percentile, *n* (%)	3 (11.1)	-	4 (14.8)
-SBP and/or DBP ≥ 95^th^ p percentile, *n* (%)	2 (7.4)	3 (11.1)	3 (11.1)
-SBP and/or DBP and/or MAP ≥ 95^th^ p percentile, *n* (%)	3 (11.1)	3 (11.1)	4 (14.8)
Blood pressure load, *n* (%)			
SBP			
≥ 50%	1 (3.7)	1 (3.7)	3 (11.1)
25–49%	3 (11.1)	2 (7.4)	1 (3.7)
DBP			
≥ 50%	-	-	-
25–49%	1 (3.7)	-	4 (14.8)
Dipping			
SBP (%)			10.7 ± 2.7
Non-dipping (< 10%), *n* (%)			9 (33.3)
DBP (%)			18.9 ± 5.0
Non-dipping (< 10%), *n* (%)			2 (7.4)

Daytime is defined as 08.00 am—08.00 pm; night-time is defined as 00.00–06.00 am

*DBP* Diastolic blood pressure, *MAP* Mean arterial pressure, *SBP* Systolic blood pressure, *P* Percentile, *SDS* Standard deviation score

### Ultrasonography

Kidney and Doppler US were performed in 71 patients. The median kidney longitudinal length (42^nd^ percentile, IQR 51^st^ percentile for the right kidney; 57^th^ percentile, IQR 48^th^ percentile for left kidney), and the mean parenchymal thickness (10.93 ± 1.52 mm [range: 8–15 mm] for the right kidney, 12.75 ± 2.25 mm [range: 9–19 mm] for the left kidney] of both kidneys were within normal limits. Only six patients (8.5%) had minor abnormal kidney US findings, including unilateral ectopic kidney (one right, one left) and minimal dilatation of the collecting system (one bilateral; three unilateral). Doppler US results were normal in all cases.

### Associated factors for long-term kidney dysfunction

**Fig. 2:**
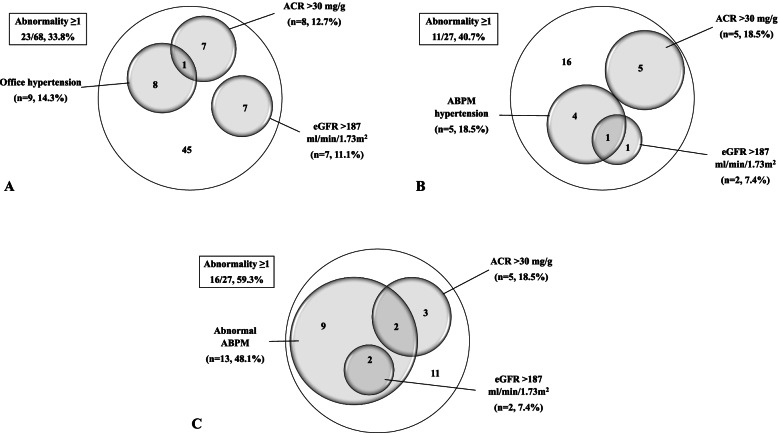
**A** Frequency of hypertension (by office blood pressure measurement), microalbuminuria and hyperfiltration among 68 patients: 23 patients (33.8%) had at least one abnormality (*N* = 68). **B** Frequency of hypertension (by ABPM), microalbuminuria and hyperfiltration among 27 patients: 11 patients (40.7%) had at least one abnormality (*N* = 27). **C** Frequency of abnormal ABPM (hypertension and/or non-dipping), microalbuminuria and hyperfiltration among 27 patients: 16 patients (59.3%) had at least one abnormality (*N* = 27). ABPM: ambulatory blood pressure monitoring, ACR: urinary albumin to creatinine ratio (mg/g), eGFR: estimated glomerular filtration rate, HT: hypertension

Office hypertension, proteinuria, and hyperfiltration were present in 13.0% (9 of 69), 12.7% (9 of 71), and 9.7% (7 of 72) of patients, respectively. None of the patients had an eGFR < 90 ml/min/1.73 m^2^. Among 68 patients, 23 patients (33.8%) had at least one sign of KDS-1; among 27 patients who had ABPM, 11 (40.7%) and 16 (59.3%) had at least one sign of KDS-2 and KDS-3, respectively (Fig. 2a, b and c).

**Fig. 3:**
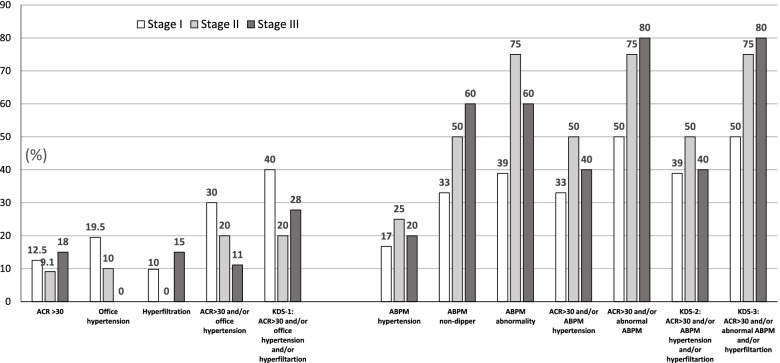
The distribution of long-term kidney dysfunction parameters according to acute kidney injury stage. ABPM: ambulatory blood pressure monitoring, ACR: urinary albumin to creatinine ratio (mg/g), KDS: kidney dysfunction set, KDS-1: presence of microalbuminuria and/or hypertension by office blood pressure and/or hyperfiltration (eGFR > 187 ml/min/1.73m^2^), KDS-2: the presence of microalbuminuria and/or hypertension with ABPM or hyperfiltration, KDS-3: the presence of microalbuminuria and/or abnormal ABPM (hypertension and/or non-dipping) or hyperfiltration

The distribution of kidney dysfunction parameters according to AKI stage groups are shown in Fig. 3. Stage II and III patients tended to have a higher rate of ABPM hypertension, ABPM non-dipper, abnormal ABPM, and KDS-3, but the differences did not reach statistical significance. Exclusion of hyperfiltration from composite outcome did not result in a significant difference.

Girls had a higher rate of microalbuminuria than boys (7 of 26, 26.9% vs. 2 of 45, 4.4%; *p* = 0.010). A higher percentage of girls also met KDS-1 criteria compared to boys (12 of 24, 50.0% vs. 11 of 44, 25.5%, *p* = 0.037). The rate of hyperfiltration (GFR > 187 ml/min/1.73 m^2^) in the large for gestational age (LGA) group was higher with a borderline significance compared to small for gestational age (SGA) and appropriate for gestational age (AGA) (3 of 9, 33.3% vs. 0 of 8, 0% and 4 of 55, 7.3%, respectively; *p* = 0.031). However, analysis using the Bonferroni method indicated that the comparisons between each paired group were not significantly different. ABPM hypertension was more frequent in patients with a birth weight less than 1,500 g (4 of 10, 40.0% in < 1,500 g group vs. 1 of 17, 5.9% in > 1,500 g group, *p* = 0.047), and in patients with a gestational age less than 32 weeks (four of 10, 40.0% in gestational age < 32 weeks group vs. one of 17, 5.9% in > 32 weeks group; *p* = 0.047). No significant association was found with other factors.

## Discussion

In our study, one-third of children who had AKI during the neonatal period had at least one sign of long-term kidney dysfunction after a mean follow-up time of 7 years, including hypertension (13.0%), proteinuria (12.7%), and hyperfiltration (9.7%). To our knowledge, this is the first study that analyzed ABPM in a cohort with neonatal AKI, and it was found to be abnormal in approximately 50% of cases.

The distribution of gestational age and birth weight were representative. Systemic diseases among the pre-renal causes and risk factors for AKI, such as sepsis, PDA, RDS, and dehydration rates, were found to be high, and these rates were similar to those found in other studies [33, 34]. It was noteworthy that only seven patients (9.7%) underwent KRT for AKI. Unlike previous studies, our study population mainly consisted of cases with Stage 1 and 2 AKI. Additionally, we excluded primary kidney diseases and diseases that could have a confounder effect on long-term kidney dysfunction. These features offered a unique opportunity to evaluate long-term outcomes in a cohort mainly with stage 1 and 2 AKI and without primary kidney, cardiac or metabolic diseases.

Routine serum biochemistry values ​​for kidney function were normal at the re-evaluation period. Abitbol et al. showed high serum creatinine and low GFR values in 9 (45%) patients in a long-term follow-up study of newborns with AKI (*n* = 20) who weighed < 1,000 g [19]. In the IRENEO prospective controlled study in children born preterm Bruel et al. showed that although there was no significant difference between the AKI group (*n* = 25) and the non-AKI group (*n* = 49), the mean GFR was lower in children with very low birth (< 1,000 g) independent of AKI [35]. Contrary to those studies, in our cohort, the mean GFR level of the nine patients with a birth weight of < 1,000 g was comparable to those with a birth weight of ≥ 1,000 g. The predominance of stage 1 and 2 AKI and the exclusion of other confounders in our cohort may be the cause of this difference.

Seven patients (9.7%) had hyperfiltration (eGFR > 187 ml/min/1.73 m^2^). There is evidence that hyperfiltration is a marker for kidney dysfunction, and these patients may be considered as stage 1 CKD [36]. There is no consensus on the definition of hyperfiltration; we defined it as a GFR > 187 ml/min/1.73 m^2^ (> mean + 2SD). In the study performed by Askenazi et al., hyperfiltration (defined as > 150 ml/min/1.73 m^2^) was present in 31% (9 out of 29 patients) of patients with AKI (age of diagnosis 6.4 ± 5.8 years) after 3–5 years follow-up [10]. Using the same definition, the prevalence of hyperfiltration was much higher (51%) in our series. Moreover, in our study, hyperfiltration was higher in patients who were LGA. Compensatory kidney growth is a characteristic adaptation to form the kidney’s normal maturation pattern during the postnatal period for patients with reduced kidney mass, as in SGA at birth [36]. Hypertrophy of tubules and glomeruli is accompanied by increased single nephron glomerular filtration rate and tubular reabsorption of sodium [36, 37]. However, none of our patients with SGA had hyperfiltration. Our results may be due to a small number of SGA and LGA patients and a lack of additional perinatal data, such as the presence of gestational diabetes.

Microalbuminuria is one of the indicators of kidney damage, and it can be seen in the early stages of CKD when changes in clinical and routine laboratory findings have not yet occurred. Microalbuminuria was identified as a long-term effect of AKI in the neonatal period in several studies, and its prevalence was 45–66% [10, 19, 35, 38]. We hypothesize that the lower prevalence of microalbuminuria in our series (12.7%) is associated with the higher percentage of stage 1 and 2 AKI compared to other series and the exclusion of confounder diseases. Additionally, in our series, the prevalence of microalbuminuria was higher in girls. In adults, lower average urine creatinine associated with lower lean body mass results in higher average ACR among females [39, 40]. In children, on the other hand, a higher prevalence of microalbuminuria in females is associated with a higher urinary albumin concentration rather than differences in urinary creatinine [39, 40]. Larkins et al. reported that the prevalence of microalbuminuria was 15.5% for girls (95% CI 10.7–20.3) and 10.2% for boys (95% CI 6.1–14.2) [41]. The greater difference in albuminuria observed in our cohort (31.4% in girls vs. 5.8% in boys) suggests that there might be additional factors that increase the risk of albuminuria in girls following neonatal AKI.

Hypertension, a well-known finding after kidney injury, was detected with office BP measurements in nine patients (9 of 69, 13%). The prevalence of hypertension in our population was shown to be 6.1% in a population based study covering 3622 children aged 5–18 years (42). One patient had isolated systolic hypertension, and eight patients had both systolic and diastolic hypertension. In other studies, examining the long-term effects of patients with AKI, the frequency of hypertension varied between 3 and 20% [12, 13, 35, 43]. Animal and human studies suggest that infants with prematurity or low birth weight (< 2,500 g) have a greater risk of developing hypertension, dyslipidemia, cardiovascular diseases and diabetes mellitus in adulthood [44, 45]. In our study, however, the presence of prematurity (14.3% vs. 11.8%, respectively) or low birth weight (< 2,500 g; 14.3% vs. 11.8%, respectively) had no significant effect on hypertension detected by office BP.

ABPM was performed in 27 patients (37.5%). Five patients (18.5%) had hypertension by ABPM. ABPM hypertension was more frequent in patients with a birth weight < 1,500 g (40.0% vs. 5.9%, *p* = 0.047), and in patients with a gestational age < 32 weeks (40.0% vs. 5.9%, *p* = 0.047). As suggested in the Brenner hypothesis, low nephron numbers or nephron deficiency, such as low birth weight, SGA, preterm birth, may increase the risk of hypertension and kidney disease in the long-term into adulthood [36]. Kwinta et al. reported that the frequency of hypertension by ABPM in children 6–7 years of age with extremely very low birth weight (< 1,000 g) was comparable to that of children in the normal birth weight group (10.8% and 5.2%, respectively; *p* = 0.5); while diastolic (27% vs. 17% *p* < 0.01) and systolic (28% vs. 16%; *p* < 0.01) BP loads were higher in the very low birth weight group [46]. In another study, low birth weight children (< 2,500 g) were compared with children with normal birth weight and 24-h and daytime diastolic BP, night systolic and diastolic BP were found to be higher in the group with low birth weight [47]. In addition, in a previous study, our group found that preterm children (5–17 years) had higher night systolic BP compared to term children (respectively 100.2 ± 7.5 mmHg and 96.3 ± 5.8 mmHg, *p* < 0.05) [48]. We hypothesize that the high prevalence of hypertension in our series may be associated with birth weight and not solely due to AKI.

The non-dipping phenomenon detected by ABPM is known to be associated with metabolic syndrome, diabetes mellitus, obesity, hypertensive end-organ damage, and cardiovascular diseases [49]. In our study, we found that 11 patients (40.7%) were systolic or diastolic non-dippers. It was noteworthy that the frequency of non-dipping was higher than the frequency of microalbuminuria, hypertension detected by office BP, hypertension detected by APBM, and hyperfiltration. The non-dipping phenomenon may be the first sign of kidney dysfunction, as seen in diabetes mellitus [50]. Although it has been shown that dipping levels at an older age are lower in children who had low birth weight and prematurity compared with control groups [49, 51], in our study, we could not identify any factor associated with the non-dipping phenomenon in newborns with AKI. Longitudinal studies in larger series through adolescence and early adulthood may be of value.

Although we did not detect any significant anomalies in our study, kidney volume loss was identified as a long-term effect of AKI in the neonatal period [18, 35, 38]. None of our patients had any structural abnormalities with kidney Doppler US. This finding may be related to the predominance of stage I and II AKI and the exclusion of patients with primary kidney diseases and other confounders.

One- to two-thirds of the patients had at least one long-term kidney dysfunction. Notably, the correlation between microalbuminuria, hyperfiltration, and hypertension was not remarkable. Longitudinal studies are needed to understand the evolution of these abnormalities, their interaction, and the significance of the non-dipping pattern.

We are aware of the limitations of our study. The cross-sectional design was the first limitation; as a result, we were not able to examine changes over time. Second, eGFR was based on serum creatinine. Third, we were not able to evaluate markers for renal tubular injury other than TPR and metabolic acidosis. Fourth, lack of a non-AKI comparison group is a limitation of the study. Although the follow-up duration in our cohort is comparable to other studies, a longer follow-up could be associated with higher prevalence of the outcomes. However, compared to previous studies, this study was strengthened by a relatively larger patient group, long-term follow (up to 12 years, median 7 years), exclusion of diseases that may interfere with long-term outcome, and performance of ABPM.

## Conclusions

In conclusion, in a cohort of patients mainly with stage 1 and 2 AKI during the neonatal period and excluding those with primary diseases that may affect the kidney outcome, we found an increased rate of office hypertension, glomerular hyperfiltration, and microalbuminuria approximately 7 years after the AKI episode. Moreover, we showed that majority of patients had abnormal ABPM pattern with a very high rate of non-dipping. Long-term and longitudinal follow-up of these patients is necessary to shed light on the significance of these parameters and the value of any intervention.

## Data Availability

The authors confirm that the data supporting the findings of this study are available within the article. The other data in this study are available from the corresponding author upon reasonable request.

## Abbreviations

AKI: Acute kidney injury
ABPM: Ambulatory blood pressure monitoring
AGA: Appropriate for gestational age
BP: Blood pressure
BMI: Body mass index
CKD: Chronic kidney disease
DBP: Diastolic blood pressure
DIC: Disseminated intravascular coagulation
ESKD: End-stage kidney disease
eGFR: Glomerular filtration rate
IQR: Interquartile range
KDIGO: Kidney Disease: Improving Global Outcomes
KDS: Kidney dysfunction set
KRT: Kidney replacement therapy
LGA: Large for gestational age
MAP: Mean arterial pressure
NEC: Necrotizing enterocolitis
NICU: Neonatal intensive care units
NSAID: Non-steroidal anti-inflammatory drug
PDA: Patent ductus arteriosus
RRI: Renal resistive index
RDS: Respiratory distress syndrome [RDS]
SD: Standard deviations
SGA: Small for gestational age
SDS: Standard deviations scores
SBP: Systolic blood pressure
TPR: Tubular phosphorus reabsorption
US: Ultrasonography
